# Trends in prevalence of hearing loss in adults in the USA 1999–2018: a cross-sectional study

**DOI:** 10.1186/s12889-024-18426-9

**Published:** 2024-04-08

**Authors:** Fengxin Mo, Shiheng Zhu, Hanlu Jia, Yuan Xia, Li Lang, Qiutong Zheng, Xiaojing Yuan, Shan Wu, Yan Bai, Wenhan Yang, Liang Wang, Qingsong Chen

**Affiliations:** 1 Department of Public Health, School of Public Health, Guangdong Pharmaceutical University, Guangzhou, Guangdong Province 510006 China; 2 Guangdong Province Hospital for Occupational Disease Prevention and Treatment, Guangzhou, Guangdong Province 510300 China; 3 Department of Public Health, Robbins College of Health and Human Sciences, Baylor University, Waco, TX 76798 USA; 4 Guangdong Provincial Engineering Research Center of Public Health Detection and Assessment, Guangdong Pharmaceutical University, Guangzhou, 510310 China

**Keywords:** Hearing loss, Prevalence, NHANES, Adults

## Abstract

**Background:**

A better understanding of how the prevalence of hearing loss and its associated factors change over time could help in developing an appropriate program to prevent the development of hearing loss.

**Methods:**

Population-representative cross-sectional data from the United States National Health and Nutrition Examination Survey (NHANES) were used to estimate the trends in the prevalence of hearing loss among adults in the USA over the period 1999–2018. A total of 15,498 adult participants aged 20 years or older had complete audiometric examination data. Logistic regression was employed to evaluate the trend in hearing loss; weighted Rao-Scott χ2 tests and univariate logistic regression analyses were used to examine the association between hearing loss and relevant factors.

**Results:**

The overall hearing loss prevalence in 1999–2018 was 19.1% 19.1 (95% CI, 18.0–20.2%). The prevalence of hearing loss decreased in cycles (*P* for trend < 0.001). For participants aged 20–69 years, the prevalence decreased from 15.6% (95% CI, 12.9–18.4%) in 1999–2000 to 14.9% (95% CI, 13.2– 16.6%) in 2015–2016; for participants aged > 70 years the prevalence decreased from 79.9% (95% CI, 76.1–83.8%) in 2005–2006 to 64.5% (95% CI, 58.8–70.2%) in 2017–2018. Participants with hearing loss were likely to be older, male, non-Hispanic white, and to have not completed high school. Mild hearing loss was more prevalent among those aged 20–79 years; in those aged over 80 years the prevalence of moderate hearing loss exceeded that of mild loss. Among all otologically normal participants, hearing thresholds increased with age across the entire frequency range.

**Conclusions:**

The prevalence of hearing loss in USA adults changed over the period 1999–2018. The trends observed provide valuable insight for making public health plans and allocating resources to hearing care. Further investigation is necessary to monitor hearing loss and its potential risk factors.

## Introduction

Hearing loss is becoming an increasingly serious public health concern in the USA. According to the estimates of Global Burden of Disease Study (GBD), more than 1.5 billion people were affected by hearing loss in 2019, amounting to 20% of the global population. Of those affected, 1.17 billion people had mild hearing loss [1]. There are potential socioeconomic impacts of hearing loss, including underemployment, reduction of income, and low educational attainment [2]. Moreover, hearing loss is the third most common cause of years lived with disability (YLDs), and is responsible for 43.5 million YLDs globally [3]. Individually, hearing loss has effects on quality of life, psychosocial health [4], and economic independence [1, 5], and affects language discrimination, resulting in communication difficulties [6], low learning and work efficiency, and a reduction in social interaction [4, 7].

The prevalence of hearing loss and the distribution of hearing threshold levels over time are due to many factors: the change in the national population structure, the diversity of the labor force, lifestyle changes, an increase in occupational noise exposure and environmental noise, the emergence of new diseases, an improvement in the awareness of hearing loss, advances in clinical medicine, and the development of new pharmaceuticals [7–9]. Studies reported in the literature have shown differences in the prevalence of hearing loss, but there has been a lack of long-term and continuous trend analysis of the prevalence of hearing loss [3, 10–12]. As most studies of hearing loss use self-report forms as a survey tool, the prevalence of mild and unilateral hearing loss may have been underestimated [13, 14].

The World Health Organization (WHO) has adopted a more standardized grading system for hearing-loss severity that is based on hearing measurements. Hearing loss is classified as mild, moderate, moderately severe, profound, and complete or total hearing loss/deafness. The new grading system has resulted in the measure of the onset of mild hearing loss being reduced from a hearing threshold of 26 dB to 20 dB, and has added the definition of unilateral hearing loss (< 20 dB in the better ear, 35 dB or greater in the worse ear) [7]. In addition to the classification, the revised system provides a description of the consequences on communication capabilities that may accompany each severity level. The new WHO hearing loss standardized grading system enables identification of asymmetric hearing loss and unilateral deafness, a more accurate description of hearing loss, and a better understanding of the difficulties in listening to the surrounding environment and speech communication in people with different levels of hearing loss. According to the WHO’s World Report on Hearing, released in 2021 [7], the prevalence across all WHO regions of moderate or high-grade hearing loss is in the range 10.9–17.6% in people aged 60–69 years, rising to 41.9–51.2% in people aged 80–89 years, and 52.9–64.9% in people aged > 90 years.

Accurate and representative data on the prevalence of hearing loss are important to understand the consequences of hearing needs, and to make plans regarding the allocation of the limited resources available for hearing healthcare [15]. It is also important that the data on the distribution of hearing threshold levels in the otologically normal population are updated from time to time. Therefore, we analyzed the periodic prevalence of hearing loss among adults in the USA aged 20 years and older using data from NHANES for the period 1999–2018, which was obtained using pure tone audiometry testing. In addition, we compared the distribution of hearing threshold levels from NHANES with those given in ISO 7029:2017 (Acoustics – Statistical distribution of hearing thresholds related to age and gender). We aimed to characterize the changes in the prevalence of hearing loss in this population from 1999 to 2018, in order to evaluate whether adult hearing loss changed over that period. A better understanding of the trends in hearing loss and its prevalence and associated factors will provide policy makers and public health researchers with more evidence that they can use when developing targeted strategies for the prevention of hearing loss.

## Methods

### Study population

NHANES is a nationally representative survey conducted by the National Center for Health Statistics (NCHS) of the Centers for Disease Control and Prevention (CDC) to assess the health and nutritional status of the civilian, non-institutionalized US population. The survey utilizes a complex, multistage, probability sampling design.

The present study used the NHANES data for the period 1999–2018. We included the participants aged 20–69 years in the cycles 1999–2004, 2011–2012, and 2015–2016, and all participants aged 70 years or older in the cycles 2005–2006, 2009–2010, and 2017–2018, because these cycles included the variables of interest.

NHANES oversampled certain populations (e.g. Mexican Americans, non-Hispanic black Americans), which allowed for increased reliability and precision of health status indicator estimates for these groups. Audiometric examinations were conducted on half of the sample of interviewed adults aged 20–69 years in 1999–2004, and all of the adults interviewed at the mobile examination center in 2005–2018 (except 2013–2014). To account for the complex sampling design and non-responses in NHANES, we applied appropriate sample weights in our analyses.

We calculated the percentile distributions of hearing threshold levels based on 3,454 otologically normal participants (exclusions: ear infection, tinnitus, non-occupational noise exposure, occupational noise exposure, and unilateral hearing loss) (NHANES data for 1999–2018) stratified by age and sex.

### Audiometric measurements

In 1999-2016, the audiometry exam sections were performed by technicians professionally trained by a certified audiologist from the National Institute for Occupational Safety and Health (NIOSH) in a dedicated sound-isolating room in the Mobile Examination Center (MEC). Instrumentation for the Audiometry Component included an Interacoustics Model AD226 audiometer with standard TDH-39 headphones and Etymotic EarTone 3A insert earphones. Audiometric calibration and background noise levels were checked using a Quest Model 1800 Precision Integrating Sound Level Meter and Model OB-300 1/3–1/1 octave filter set. Daily monitoring of calibration and ambient noise levels was accomplished with a Quest Model BA-201–25 Bioacoustic Simulator and Octave Band Monitor. The audiometers used in this survey met the specifications of ANSI S3.6–1996 for Type 3 audiometers. Hearing threshold testing was conducted on both ears of subjects at seven frequencies (500, 1000, 2000, 3000, 4000, 6000, and 8000 Hz), and the testing threshold range was from –10 to 120 dB. Subjects using hearing aids who were not able to remove them for testing and subjects who had so much ear pain at the time of the examination that they could not tolerate headphones were excluded from the audiometry component of the survey. There were no other precluding conditions for any part of the audiology examination.

Beginning in the 2017–2018, the ART system was used to conduct the pure-tone air conduction hearing test on survey participants. The ART system is a comprehensive, high configurable audiometer designed for the research application. Instrumentation for the audiometry component included an Audiometric Research Tool (ART) system with standard TDH-49P headphones and Etymotic EarTone 3A insert earphones. Daily monitoring ambient noise levels was accomplished with a Quest Model BA-202-27 Bioacoustic Simulator.AUX data were entered directly into the computerized NHANES database system. Data from the ART system and Interacoustics Titan were captured electronically and uploaded into the survey information system automatically.

### Definition of hearing loss

Hearing thresholds are usually measured by pure-tone audiometry, which estimates the minimum sound intensity of pure tones at a range of frequencies. The pure-tone average (PTA) is the average of the hearing-threshold level at frequencies of 500, 1000, 2000, and 4000 Hz in the better ear. The better ear was defined as the ear showing the lower PTA. Hearing loss was categorized according to the WHO classification: normal hearing(less than 20 dB), mild (20 to < 35 dB), moderate (35 to < 50 dB), moderately severe (50 to < 65 dB), severe (65 to < 80 dB), profound (80 to < 95 dB), and complete or total hearing loss/deafness (95 dB or greater); and unilateral hearing loss (< 20 dB in the better ear, 35 dB or greater in the worse ear) [7]. Since the numbers of subjects in the more refined categories were very small, severe hearing loss, profound hearing loss, and complete or total hearing loss/deafness were merged into the moderately severe hearing loss group.

### Demographic variables

Information about age, sex, race/ethnicity, educational level, and family income/poverty level ratio was collected during the NHANES in-person interviews. Participants were grouped into 10-year categories by age. Race and ethnicity were categorized as Hispanic (including Mexican American and other Hispanic), non-Hispanic white, non-Hispanic black, and other (including non-black, non-Hispanic groups such as Asian-American, Native American, and mixed race). Educational level was grouped into the following categories: less than high school (including less than 9th grade, 9–11th grade (includes 12th grade with no diploma)), high school (high school graduate/GED or equivalent), and college or higher (including some college or AA degree, college graduate or above). Family income/poverty level ratio was used the poverty/income ratio (PIR), which was defined as the total family income divided by the poverty threshold, as determined by the US Census Bureau, for the year of the interview. It was grouped into the following categories: low (PIR ≤ 1.3), middle (1.3 < PIR ≤ 3.5), and high (PIR > 3.5).

### Relevant variables

The occupational noise exposure in the NHANES cycles for 1999–2004 was defined as exposure to loud noise in the “current job.” In the 2005–2010 cycles, participants were asked whether they were exposed to job-related loud noise for at least 5 h per week. In the 2011–2012 and 2015–2018 cycles, participants were asked if they had ever had a job or a combination of two jobs that exposed them to loud noise for at least 4 h a day or on several days a week. We grouped the answers as “yes” and “no” (which included “no” and “never worked”) for the 2011–2012 and 2015–2018 cycles.

The non-occupational noise exposure in the 1999–2004 cycles was defined as exposure to noise at least once a month for a year outside work. In the 2005–2010 cycles, participants were asked whether they were exposed to noise for 5 h or more per week outside work. “At least 10 h per week in non-occupational noise exposure” was defined as exposure in the 2011–2012 and 2015–2018 cycles.

We used the results from the survey self-assessment questionnaire to identify the presence of tinnitus and/or ear infection. We grouped the answers as “yes” and “no”.

### Statistical analyses

All statistical analyses were performed by considering the weights necessary due to the complex sampling design and according to the guidelines for the analysis of NHANES data. The weighted prevalences and 95% CIs of hearing loss were estimated. The demographic sample proportions were compared using the Rao-Scott χ^2^ tests from the survey. The survey univariate logistic regression was used to evaluate the trend in hearing loss. We provided percentile distributions of hearing threshold levels, stratified by age and sex. The relevant variables are used to show the stratified trends. All data analyses were performed using SAS 9.4 (SAS Institute, Cary, North Carolina, USA).

### Ethics

NHANES has been approved by the NCHS Ethics Review Board. Data are released in 2-year cycles and are made publicly available online. Written informed consent is obtained from all survey participants. The Guangdong Pharmaceutical University Academic Review Board determined that the present study was exempt from approval because of the use of de-identified data.

## Results

### Baseline characteristics

Tables 1 and 2 shows the characteristics of survey participants according to the auditory threshold test in the 1999–2018 cycles of NHANES. A total of 15,498 individuals were included over 8 cycles (those without complete hearing threshold test data or aged under 20 years were excluded). Table is divided into Tables 1 and 2.

**Table 1:** Characteristics of participants in NHANES according to the auditory threshold by cycle (1999–2006)

Participants, *N* (%)^a^												
	1999–2000			2001–2002			2003–2004			2005–2006		
Characteristic	Normal hearing	Hearing loss	***P* value**^**b**^	Normal hearing	Hearing loss	***P* value**^**b**^	Normal hearing	Hearing loss	***P* value**^**b**^	Normal hearing	Hearing loss	***P* value**^**b**^
Overall	1346	309		1551	310		1462	288		120	543	
Age, *N* (%),			< 0.001			< 0.001			< 0.001			< 0.001
20–29 years	378	5		424	12		405	11		-^c^	-^c^	
	25.6 (20.2–31.1)	1.9 (0.0–4.1)		23.9 (20.8–27.1)	3.6 (0.8–6.4)		24.5 (22.3–26.7)	5.1 (0.3–9.9)				
30–39 years	330	18		366	26		353	15		-^c^	-^c^	
	28.9 (24.0–33.8)	8.2 (4.8–11.6)		25.1 (20.9–29.3)	9.6 (3.8–15.5)		25.8 (23.0–28.6)	5.8 (2.2–9.5)				
40–49 years	267	38		363	45		309	39		-^c^	-^c^	
	23.4 (19.8–27.0)	16.3 (9.3–23.2)		28.2 (24.5–31.8)	19.8 (15.7–23.8)		25.7 (22.9–28.6)	17.7 (12.2–23.2)				
50–59 years	187	74		231	87		204	75		-^c^	-^c^	
	14.5 (11.7–17.2)	28.7 (18.4–39.0)		16.3 (13.4–19.2)	35.7 (29.6–41.8)		16.3 (13.5–19.1)	36.7 (29.5–43.8)				
60–69 years	184	174		167	140		191	148		-^c^	-^c^	
	7.6 (6.0–9.3)	45.0 (34.5–55.5)		6.5 (5.1–7.8)	31.3 (25.5–37.1)		7.7 (6.3–9.1)	34.7 (28.8–40.7)				
70–79 years	-^c^	-^c^		-^c^	-^c^		-^c^	-^c^		101	301	
										87.0 (80.3–93.6)	62.9 (58.3–67.6)	
80 + years	-^c^	-^c^		-^c^	-^c^		-^c^	-^c^		19	242	
										13.0 (6.4–19.7)	37.1 (32.4–41.7)	
Sex, *N* (%)			< 0.001			< 0.001			< 0.001			< 0.001
Men	580	204		655	209		651	173		47	294	
	47.2 (43.4–51.0)	67.1 (61.0–73.1)		43.4 (40.0–46.8)	68.8 (61.9–75.6)		46.6 (43.5–49.8)	60.0 (55.7–64.3)		28.7 (22.6–34.7)	43.6 (38.9–48.3)	
Women	766	105		896	101		811	115		73	249	
	52.8 (49.0–56.6)	32.9 (26.9–39.0)		56.6 (53.2–60.0)	31.2 (24.4–38.1)		53.4 (50.2–56.2)	40.0 (35.7–44.3)		71.3 (65.3–77.4)	56.4 (51.7–61.1)	
Race, *N* (%)			0.21			0.003			< 0.001			0.36
Hispanic	475	113		428	72		341	76		8	60	
	17.9 (11.1–24.6)	15.7 (5.6,25.9)		13.8 (8.2–19.5)	9.5 (4.6–14.4)		12.7 (7.1–18.3)	10.0 (4.9–15.1)		2.6 (0.3–4.8)	4.2 (2.6–5.7)	
Non-Hispanic white	573	139		727	170		718	161		79	409	
	66.9 (60.8–73.0)	73.8 (62.7–84.8)		68.7 (63.2–74.3)	77.5 (70.2–84.8)		69.5 (62.2–76.8)	77.3 (69.6–85.1)		85.2 (76.2–94.2)	87.9 (83.8–92.1)	
Non-Hispanic black	253	49		329	57		336	35		30	68	
	11.3 (7.8–14.7)	7.3 (2.6–12.0)		11.9 (7.9–15.9)	8.3 (3.6–13.0)		12.7 (8.6–16.8)	6.0 (2.8–9.2)		10.3 (2.8–17.8)	6.6 (3.7–9.6)	
Other	45	8		67	11		67	16		3	6	
	4.0 (1.9–6.1)	3.2 (0.0–7.4)		5.6 (3.9–7.2)	4.7 (1.5–7.8)		5.1 (3.5–6.7)	6.7 (2.9–10.5)		1.9 (0.0–4.5)	1.3 (0.0–2.8)	
Educational level, *N* (%)			< 0.001			< 0.001			< 0.001			0.02
Less than high school	429	153		396	113		330	106		30	216	
	20.0 (17.0–22.9)	36.9 (30.2–43.6)		15.8 (13.4–18.3)	25.2 (18.3–32.1)		14.2 (12.4–16.0)	22.4 (13.7–31.1)		18.8 (9.0–28.6)	32.1 (26.4–37.9)	
High school	302	69		342	65		336	86		34	156	
	23.8 (20.7–27.0)	28.5 (23.2–33.8)		23.9 (21.1–26.8)	25.9 (22.0–29.8)		24.3 (21.0–27.6)	35.4 (25.8–45.0)		28.7 (17.3–40.0)	32.8 (27.7–37.8)	
College or higher	615	87		813	132		796	96		56	171	
	56.2 (51.8–60.5)	34.6 (28.4–40.8)		60.2 (55.9–64.5)	48.9 (43.5–54.4)		61.5 (59.0–63.9)	42.2 (33.7–50.7)		52.5 (37.0–68.0)	35.1 (26.8–43.4)	
Poverty/income ratio, *N* (%)			0.206			0.255			0.060			0.002
Low	327	82		387	68		383	94		20	143	
	20.3 (14.3–26.2)	18.8 (8.9–28.8)		19.4 (17.7–21.1)	16.0 (10.0–22.0)		18.8 (14.7–23.0)	23.5 (15.6–31.4)		12.4 (6.9–17.9)	20.8 (16.8–24.8)	
Middle	419	100		516	117		497	105		53	255	
	29.2 (24.0–34.5)	32.9 (23.8–41.9)		30.1 (27.4–32.8)	33.5 (25.4–41.7)		33.9 (28.4–39.5)	37.7 (30.9–44.5)		43.4 (33.5–53.3)	50.0 (43.8–56.2)	
High	411	72		553	99		503	71		41	104	
	40.2 (33.5–46.9)	32.5 (18.0–47.0)		44.5 (40.6–48.4)	42.0 (32.8–51.2)		42.0 (36.4–47.6)	32.6 (22.9–42.2)		37.2 (24.5–49.8)	21.4 (16.4–26.4)	
Missing	189	54		95	26		79	18		6	41	
	10.3 (6.5–14.1)	15.8 (6.7–25.0)		6.0 (3.5–8.6)	8.5 (4.9–12.1)		5.3 (3.0–7.5)	6.3 (3.8–8.7)		7.1 (0.6–13.5)	7.8 (4.7–10.8)	
Non-occupational noise exposure, *N* (%)			0.18			0.10			0.49			< 0.001
Yes	267	49		364	84		393	72		8	97	
	23.1 (19.7–26.4)	19.5 (14.9–24.2)		26.6 (23.5–29.6)	34.0 (24.2–43.9)		29.5 (26.5–32.5)	27.2 (20.6–33.8)		5.0 (1.4–8.5)	15.6 (11.6–19.6)	
No	1079	260		1187	226		1069	216		112	446	
	76.9 (73.6–80.3)	80.5 (75.8–85.1)		73.4 (70.4–76.5)	66.0 (56.1–75.8)		70.5 (67.5–73.5)	72.8 (66.2–79.5)		95.0 (91.5–98.6)	84.4 (80.4–88.4)	
Occupational noise exposure, *N* (%)			0.002			0.11			0.35			0.02
Yes	140	39		173	46		151	32		32	229	
	11.5 (8.2–14.8)	16.7 (9.9–23.5)		12.0 (9.2–14.8)	18.8 (11.8–25.7)		11.7 (9.0–14.5)	14.4 (8.7–20.1)		20.2 (10.7–29.7)	37.2 (31.7–42.8)	
No	135	20		172	38		174	25		88	314	
	12.3 (9.9–14.6)	5.6 (1.8–9.5)		13.0 (11.1–15.0)	14.2 (9.0–19.4)		14.1 (11.7–16.6)	11.5 (7.0–16.1)		79.8 (70.3–89.3)	62.8 (57.2–68.3)	
Missing	1071	250		1206	226		1137	231		-^c^	-^c^	
	76.2 (72.8–79.6)	77.7 (70.9–84.4)		75.0 (72.4–77.5)	67.0 (58.1–76.0)		74.2 (70.2–78.1)	74.0 (68.3–79.8)				

^a^The estimates of the percentage of participants were weighted

^b^*P* values for overall differences in prevalence by stratum

^c^No participants were observed in the sample

**Table 2:** Characteristics of participants in NHANES according to the auditory threshold by cycle (2009–2018)

Participants, *N* (%)^a^												
	2009–2010			2011–2012			2015–2016			2017–2018		
Characteristic	Normal hearing	Hearing loss	*P* value^b^	Normal hearing	Hearing loss	*P* value^b^	Normal hearing	Hearing loss	*P* value^b^	Normal hearing	Hearing loss	*P* value^b^
Overall	187	685		3285	533		3589	674		202	414	
Age, *N* (%)			< 0.001			< 0.001			< 0.001			< 0.001
20–29 years	-^c^	-^c^		828	12		840	14		-^c^	-^c^	
				24.8 (19.6–30.0)	2.4 (0.7–4.0)		24.4 (22.1–26.7)	1.8 (0.6–3.1)				
30–39 years	-^c^	-^c^		731	26		838	28		-^c^	-^c^	
				21.5 (18.6–24.4)	3.8 (2.0–5.6)		22.8 (20.3–25.2)	4.6 (2.0–7.1)				
40–49 years	-^c^	-^c^		671	67		787	73		-^c^	-^c^	
				22.8 (19.9–25.6)	14.4 (9.1–19.7)		21.9 (19.7–24.1)	12.6 (9.7–15.4)				
50–59 years	-^c^	-^c^		616	156		644	195		-^c^	-^c^	
				19.8 (17.3–22.3)	36.3 (30.0–42.7)		19.1 (17.2–21.1)	33.7 (28.3–39.2)				
60–69 years	-^c^	-^c^		439	272		480	364		-^c^	-^c^	
				11.1 (8.9–13.3)	43.1 (37.3–48.9)		11.8 (9.8–13.8)	47.3 (41.9–52.7)				
70–79 years	163	397		-^c^	-^c^		-^c^	-^c^		175	260	
	87.4 (80.8–94.0)	58.4 (55.0–61.7)								89.0 (82.4–95.5)	67.9 (62.7–73.0)	
80 + years	24	288		-^c^	-^c^		-^c^	-^c^		27	154	
	12.6 (6.0–19.2)	41.6 (38.3–45.0)								11.0 (4.5–17.6)	32.1 (27.0–37.3)	
Sex, *N* (%)			0.002			< 0.001			< 0.001			0.65
Male	59	349		1601	343		1606	430		78	192	
	26.5 (17.9–35.2)	43.2 (39.4–47.1)		47.4 (45.2–49.7)	65.6 (58.7–72.4)		46.2 (45.0–47.5)	64.2 (58.7–69.7)		35.9 (28.4–43.3)	38.1 (31.7–44.5)	
Female	128	336		1684	190		1983	244		124	222	
	73.5 (64.8–82.1)	56.8 (52.9–60.6)		52.6 (50.3–54.8)	34.4 (27.6–41.3)		53.8 (52.5–55.0)	35.8 (30.3–41.3)		64.1 (56.7–71.6)	61.9 (55.5–68.3)	
Race, *N* (%)			< 0.001			< 0.001			< 0.001			0.66
Hispanic	33	83		653	120		1098	262		31	53	
	8.2 (1.2–15.2)	5.7 (1.9–9.4)		14.8 (9.5–20.2)	10.4 (5.1–15.7)		16.6 (10.7–22.4)	13.7 (7.4–20.1)		8.7 (5.1–12.4)	7.6 (3.9–11.2)	
Non-Hispanic white	101	497		1114	213		1051	222		93	232	
	71.8 (63.8–79.8)	85.2 (80.3–90.1)		64.9 (56.4–73.3)	75.8 (67.7–83.8)		61.4 (52.8–69.9)	70.8 (61.9–79.7)		74.0 (68.0–79.9)	78.1 (70.0–86.3)	
Non-Hispanic black	41	82		936	123		834	107		51	77	
	13.4 (7.7–19.2)	6.5 (3.9–9.2)		12.4 (7.4–17.4)	7.0 (2.9–11.1)		12.4 (7.5–17.3)	7.3 (3.6–10.9)		9.4 (5.3–13.4)	7.9 (3.7–12.1)	
Other	12	23		582	77		606	83		27	52	
	6.6 (3.6–9.6)	2.6 (1.2–4.0)		7.9 (5.5–10.3)	6.9 (4.2–9.5)		9.6 (7.0–12.3)	8.2 (4.7–11.8)		7.9 (3.1–12.7)	6.4 (2.5–10.3)	
Educational level, *N* (%)			0.07			< 0.001			0.04			0.002
Less than high school	57	228		576	159		700	226		27	101	
	21.3 (13.2–29.4)	28.3 (21.9–34.7)		13.0 (9.6–16.5)	18.5 (12.9–24.1)		12.5 (9.2–15.8)	18.4 (11.7–25.0)		7.0 (3.3–10.6)	15.4 (12.2–18.5)	
High school	37	171		666	137		774	150		47	106	
	23.6 (17.7–29.6)	26.7 (22.0–31.4)		18.5 (15.4–21.6)	27.1 (21.4–32.7)		19.9 (17.7–22.2)	18.8 (12.5–25.1)		26.5 (19.0–34.0)	29.6 (22.4–36.7)	
College or higher	93	286		2043	237		2115	298		128	207	
	55.0 (44.2–65.8)	45.0 (38.4–51.7)		68.5 (62.6–74.4)	54.4 (46.2–62.6)		67.6 (62.7–72.6)	62.8 (55.3–70.3)		66.5 (60.4–72.7)	55.0 (47.8–62.3)	
Poverty/income ratio, *N* (%)			0.200			0.263			0.444			0.436
Low	37	159		1072	182		995	229		33	73	
	14.6 (8.5–20.7)	16.8 (13.5–20.1)		23.2 (18.7–27.8)	20.5 (15.8–25.1)		19.1 (15.8–22.3)	18.5 (13.6–23.4)		9.1 (6.0–12.3)	10.9 (7.8–14.0)	
Middle	78	308		1004	160		1291	231		83	190	
	41.6 (31.5–51.8)	48.2 (42.0–54.4)		30.9 (26.3–35.5)	35.1 (26.4–43.8)		33.7 (30.2–37.1)	29.9 (23.0–36.8)		38.3 (25.8–50.8)	42.2 (31.9–52.6)	
High	48	165		988	142		985	153		61	95	
	31.9 (24.0–39.9)	28.0 (21.6–34.5)		40.9 (33.8–47.9)	36.9( 28.2–45.6)		40.5 (35.3–45.7)	45.0 (34.6–55.4)		42.8 (29.6–56.1)	33.8 (22.6–45.0)	
Missing	24	53		221	49		318	61		25	56	
	11.8 (6.3–17.3)	7.0 (4.6–9.3)		5.0 (3.3–6.7)	7.5 (3.0–12.0)		6.8 (5.3–8.2)	6.6 (3.4–9.7)		9.7 (2.8–16.7)	13.0 (8.2–17.8)	
Non-occupational noise exposure, *N* (%)			0.31			0.38			< 0.001			0.08
Yes	15	79		396	72		474	137		9	39	
	7.6 (2.3–12.9)	10.3 (5.6–14.9)		12.3 (10.2–14.4)	15.0 (9.8–20.2)		14.7 (12.8–16.6)	24.6 (20.2–29.1)		4.6 (0.4–8.8)	8.4 (5.5–11.3)	
No	172	606		2889	461		3115	537		193	375	
	92.4 (87.1–97.7)	89.7 (85.1–94.4)		87.7 (85.6–89.8)	85.0 (79.8–90.2)		85.3 (83.4–87.2)	75.4 (70.9–79.8)		95.4 (9.2–99.6)	91.6 (88.7–94.5)	
Occupational noise exposure, *N* (%)			< 0.001			< 0.001			< 0.001			0.12
Yes	31	228		1064	242		1104	293		53	157	
	13.1 (8.1–18.1)	31.0 (26.8–35.1)		33.0 (29.0–37.0)	51.7 (42.0–61.4)		31.1 (28.1–34.1)	43.7 (38.9–48.6)		23.7 (14.3–33.0)	32.1 (25.1–39.0)	
No	156	457		2221	291		2485	381		149	257	
	86.9 (81.9–91.9)	69.0 (64.9–73.2)		67.0 (63.0–71.0)	48.3 (38.6–58.0)		68.9 (65.9–71.9)	56.3 (51.4–61.1)		76.3 (67.0–85.7)	67.9 (61.0–74.9)	
Missing	-^c^	-^c^		-^c^	-^c^		-^c^	-^c^		-^c^	-^c^	

^a^The estimates of the percentage of participants were weighted

^b^*P* values for overall differences in prevalence by stratum

^c^No participants were observed in the sample

### Prevalence of and trend in hearing loss in the population

The prevalence of hearing loss in the sample overall was 19.1% (95% CI, 18.0–20.2%). It was significantly higher in the elderly, those with lower educational levels, and those who experienced noise exposure. Among people aged 20–69 years, the prevalence decreased from 15.6% (95% CI, 12.9–18.4%) in 1999–2000 to 14.9% (95% CI, 13.2–16.6%) in 2015–2016; among those aged 70 years or more it decreased from 79.9% (95% CI, 76.1–83.8%) in 2005–2006 to 64.5% (95% CI, 58.8–70.2%) in 2017–2018. However, the prevalence of hearing loss increased with age within the same survey cycle. The prevalence of hearing loss decreased significantly in the age groups 30–39, 60–69, 70–79, and 80 years and over (all *P* for trend < 0.05). A significant increase in the prevalence of hearing loss was observed in women, non-Hispanic whites, high school and college or higher educational level subgroups, middle PIR and high PIR subgroups, and those experiencing non-occupational noise exposure (all *P* for trend < 0.05). Detailed subgroup results are shown in Table 3.

**Table 3:** Trends of the prevalence of hearing loss among the US adult population by cycle

Variables	Overall	1999–2000	2001–2002	2003–2004	2005–2006	2009–2010	2011–2012	2015–2016	2017–2018	*P* for trend^a^
		1	2	3	4	6	7	8	9	
No. of participants overall	15498	1655	1861	1750	663	872	3818	4263	616	
No. of participants with hearing loss	3756	309	310	288	543	685	533	674	414	
Overall prevalence^b^	19.1 (18.0–20.2)	15.6 (12.9–18.4)	15.3 (12.7–17.8)	14.8 (12.3–17.2)	79.9 (76.1–83.8)	79.1 (72.7–85.5)	14.2 (12.3–16.2)	14.9 (13.2–16.6)	64.5 (58.8–70.2)	< 0.001
Age, *N* (%)										
20–29 years	2929	5	12	11	-^c^	-^c^	12	14	-^c^	0.29
		1.4 (0.0–2.8)	2.6 (0.7–4.6)	3.5 (0.2–6.7)			1.6 (0.6–2.6)	1.3 (0.5–2.1)		
30–39 years	2731	18	26	15	-^c^	-^c^	26	28	-^c^	0.03
		5.0 (3.1–6.9)	6.5 (2.8–10.1)	3.8 (1.4–6.1)			2.8 (1.6–4.0)	3.4 (1.7–5.1)		
40–49 years	2659	38	45	39	-^c^	-^c^	67	73	-^c^	0.17
		11.4 (7.6–15.2)	11.2 (8.1–14.4)	10.7 (7.5–13.8)			9.5 (6.3–12.7)	9.1 (7.0–11.2)		
50–59 years	2469	74	87	75	-^c^	-^c^	156	195	-^c^	0.18
		26.9 (16.4–37.4)	28.2 (21.8–34.6)	28.1 (20.9–35.2)			23.3 (19.1–27.5)	23.6 (19.1–27.0)		
60–69 years	2559	174	140	148	-^c^	-^c^	272	364	-^c^	0.02
		52.3 (41.9–62.6)	46.6 (38.2–55.0)	44.0 (39.4–48.5)			39.2 (32.6–45.8)	41.2 (36.0–46.5)		
70–79 years	1397	-^c^	-^c^	-^c^	301	397	-^c^	-^c^	260	< 0.001
					74.2 (68.4–80.0)	74.2 (63.3–80.1)			58.1 (51.4–64.8)	
80 + years	754	-^c^	-^c^	-^c^	242	288	-^c^	-^c^	154	0.03
					91.9 (89.3–94.5)	92.6 (88.1–97.1)			84.1 (77.1–91.2)	
Sex, *N* (%)										
Male	7471	204	209	173	294	349	343	430	192	0.10
		20.9 (17.2–24.5)	22.2 (17.7–26.7)	18.2 (14.4–22.1)	85.8 (82.2–89.4)	86.1 (80.6–91.6)	18.6 (15.2–22.1)	19.5 (17.5–21.6)	65.9 (59.1–72.7)	
Female	8027	105	101	115	249	336	190	244	222	< 0.001
		10.4 (7.3–13.4)	9.0 (6.8–11.3)	11.5 (9.9–13.1)	75.9 (70.7–81.1)	74.5 (66.2–82.9)	9.8 (8.0–11.6)	10.4 (8.3–12.6)	63.7 (55.9–71.5)	
Race, *N* (%)										
Hispanic	3906	113	72	76	60	83	120	262	53	0.29
		14.0 (10.3–17.8)	11.0 (9.0–13.0)	12.0 (8.0–16.0)	86.5 (78.7–94.3)	72.5 (60.3–84.7)	10.4 (8.4–12.4)	12.7 (10.4–14.9)	61.1 (51.4–70.8)	
Non-Hispanic white	6499	139	170	161	409	497	213	222	232	< 0.001
		17.0 (13.4–20.5)	16.9 (13.7–20.1)	16.2 (13.8–18.5)	80.4 (75.8–85.1)	81.8 (74.9–88.7)	16.2 (14.1–18.4)	16.8 (14.7–18.9)	65.7 (59.3–72.2)	
Non-Hispanic black	3408	49	57	35	68	82	123	107	77	0.09
		10.8 (6.5–15.0)	11.2 (7.4–15.1)	7.6 (5.7–9.5)	71.9 (63.3–80.5)	64.8 (56.3–73.3)	8.5 (6.1–10.9)	9.3 (8.1–10.5)	60.5 (51.2–69.8)	
Other	1685	8	11	16	6	23	77	83	52	0.53
		12.8 (1.5–24.1)	13.1 (9.3–17.0)	18.6 (8.7–28.4)	73.0 (73.0–73.0)	59.9 (52.0–67.8)	12.7 (8.6–16.7)	13.0 (9.4–16.6)	59.5 (50.1–68.9)	
Educational level, *N* (%)										
Less than high school	3847	153	113	106	216	228	159	226	101	0.08
		25.5 (20.5–30.6)	22.3 (17.1–27.4)	21.4 (14.3–28.5)	87.2 (83.1–91.2)	83.4 (77.0–89.8)	19.0 (15.6–22.5)	20.5 (16.6–24.3)	80.0 (72.0–88.1)	
High school/GED or equivalent	3478	69	65	86	156	171	137	150	106	< 0.001
		18.1 (14.9–21.4)	16.3 (12.7–19.9)	20.2 (16.8–23.6)	82.0 (75.4–88.6)	81.1 (72.7–89.4)	19.5 (16.1–23.0)	14.2 (10.4–18.0)	67.0 (58.6–75.4)	
College or higher	8173	87	132	96	171	286	237	298	207	< 0.001
		10.3 (7.8–12.7)	12.8 (10.2–15.3)	10.6 (7.5–13.8)	72.7 (64.2–81.2)	75.6 (68.2–83.0)	11.6 (9.5–13.8)	14.0 (11.8–16.2)	60.1 (53.0–67.1)	
Poverty/income ratio, *N* (%)										
Low	4284	82	68	94	143	159	182	229	73	0.15
		14.7(9.4–20.0)	12.9 (9.9–15.9)	17.8 (13.5–22.1)	87.0 (84.1–89.9)	81.4 (72.0–90.8)	12.7 (10.4–15.0)	14.5 (12.1–16.9)	68.4 (61.2–75.6)	
Middle	5407	100	117	105	255	308	160	231	190	0.03
		17.2 (12.2–22.3)	16.7 (11.8–21.6)	16.1 (12.0–20.3)	82.1 (77.3–86.9)	81.4 (75.3–87.5)	15.8 (11.6–20.0)	13.5 (10.4–16.5)	66.7 (55.9–77.6)	
High	4492	73	99	71	104	165	142	153	95	< 0.001
		13.0 (8.4–17.6)	14.5 (11.3–17.8)	11.9 (8.6–15.1)	69.6 (61.7–77.6)	76.9 (68.3–85.4)	13.0 (10.1–16.0)	16.3 (13.7–18.8)	58.9 (49.2–68.6)	
Missing	1315	54	26	18	41	53	49	61	56	0.19
		22.1 (14.2–30.1)	20.3 (13.8–26.8)	17.1 (11.0–23.1)	81.4 (74.4–88.4)	69.1 (54.2–84.1)	19.9 (11.7–28.1)	14.6 (8.5–20.6)	70.8 (55.8–85.9)	
Non-occupational noise exposure, *N* (%)										
No	12943	260	226	216	446	606	461	537	375	0.001
		16.2 (13.4–19.1)	13.9 (11.1–16.7)	15.2 (12.3–18.1)	78.0 (73.6–82.3)	78.6 (72.8–84.5)	13.8 (11.9–15.8)	13.4 (11.5–15.3)	63.6 (57.5–69.6)	
Yes	2555	49	84	72	97	79	72	137	39	< 0.001
		13.6 (9.4–17.8)	18.8 (13.6–23.9)	13.8(10.4–17.2)	92.6 (87.4–97.7)	83.6 (70.6–96.6)	16.8 (10.3–23.3)	22.7 (20.3–25.2)	76.8 (67.4–86.1)	
Occupational noise exposure, *N* (%)										
No	7363	20	38	25	314	457	291	381	257	0.49
		7.8 (3.2–12.5)	16.4 (10.8–22.0)	12.4 (8.0–16.8)	75.8 (69.0–82.6)	75.0 (68.1–81.9)	10.7 (8.9–12.4)	12.5 (10.5–14.5)	61.8 (54.0–69.6)	
Yes	4014	39	46	32	229	228	242	293	157	0.59
		21.2 (16.7–25.7)	22.0 (13.2–30.8)	17.6 (11.1–24.0)	88.0 (83.5–92.5)	89.9 (85.2–94.7)	20.6 (16.9–24.4)	19.8 (17.4–22.2)	71.1 (62.8–79.5)	
Missing	4121	250	226	231	-^c^	-^c^	-^c^	-^c^	-^c^	0.59
		15.9 (12.5–19.3)	13.9 (11.0–16.8)	14.8 (12.3–17.2)						

^a^*P* values for trends were calculated using weighted logistic regression models, which included the survey cycle as a continuous variable

^b^Estimates of the percentage of participants were weighted

^c^No participants were observed in the sample

### Prevalence of hearing loss by severity and unilateral hearing loss in the population

Table 4 shows the prevalence of hearing loss according to the severity of hearing loss by age and cycle. The prevalence of hearing-loss severity (mild hearing loss, moderate hearing loss, and moderately severe hearing loss) increased statistically significantly by cycle. The most prevalent type of hearing loss was mild (20 to < 35 dB). The prevalence of mild hearing loss was 12.0 to 45.1% and moderate hearing loss was 1.8 to 29.0% in each survey cycle.

**Table 4:** Estimated prevalence of hearing loss grade in the US adult population

	Unilateral hearing loss	Mild hearing loss	Moderate hearing loss	Moderately severe hearing loss
No. of participants overall	208	2623	852	281
Participants, *N* (%)	1.3 (1.0–1.5)	14.4 (13.6–15.2)	3.6 (3.2–4.0)	1.0 (0.8–1.3)
Cycle				
1999–2000	22	247	49	13
	1.1 (0.5–1.7)	12.9 (11.0–14.8)	2.3 (1.0–3.5)	0.5 (0.1–0.9)
2001–2002	16	248	49	13
	1.1 (0.7–1.5)	12.4 (10.8–13.9)	2.4 (1.4–3.4)	0.5 (0.2–0.8)
2003–2004	30	240	37	11
	1.5 (0.8–2.2)	12.3 (10.2–14.5)	2.0 (1.1–2.9)	0.4 (0.0–0.9)
2005–2006	2	291	172	80
	0.3 (0.0–0.8)	45.1 (41.4–48.8)	23.7 (20.8–26.6)	11.0 (7.0–15.2)
2009–2010	6	351	245	89
	0.8 (0.3–1.2)	40.8 (37.0–44.7)	29.0 (24.4–33.5)	9.2 (7.5–11.1)
2011–2012	54	439	76	18
	1.3 (0.9–1.7)	12.0 (10.4–13.6)	1.8 (1.2–2.4)	0.5 (0.1–0.8)
2015–2016	70	549	102	23
	1.5 (1.0–1.9)	12.3 (10.7–13.9)	2.1 (1.6–2.6)	0.5 (0.2–0.8)
2017–2018	8	258	122	34
	1.1 (0.1–2.1)	40.8 (36.5–45.1)	18.0 (13.8–22.1)	5.8 (2.7–8.8)
*P* for trend	0.401	< 0.001	< 0.001	0.003
Age, *N* (%)				
20–29 years	22	50	2	2
	0.7 (0.2–1.2)	2.0 (1.1–2.8)	0.1 (0.0–0.2)	0.0 (0.0–0.1)
30–39 years	28	95	16	2
	1.0 (0.5–1.5)	3.7 (2.8–4.6)	0.6 (0.1–1.0)	0.1 (0.0–0.2)
40–49 years	33	229	25	8
	1.2 (0.6–1.9)	8.9 (7.7–10.2)	1.2 (0.5–1.9)	0.3 (0.0–0.5)
50–59 years	60	500	70	17
	1.9 (1.3–2.6)	22.3 (19.9–24.8)	2.8 (1.8–3.7)	0.7 (0.3–1.2)
60–69 years	49	849	200	49
	1.8 (1.1–2.6)	33.9 (31.0–36.7)	8.3 (6.8–9.9)	1.8 (1.0–2.6)
70–79 years	13	618	260	80
	0.9 (0.3–1.4)	44.6 (41.8–47.4)	17.4 (14.6–20.2)	5.4 (3.7–7.0)
> 80 years	3	282	279	123
	0.4 (0.0–0.9)	36.7 (33.2–40.3)	37.2 (33.7–40.8)	16.0 (12.6–19.5)
*P* for trend	0.01	< 0.001	< 0.001	< 0.001

*P* values for trends were calculated using weighted logistic regression models, which included the survey cycle as a continuous variable

There was a statistically significant increasing trend in the prevalence of hearing-loss severity by age (all *P* for trend < 0·001). The prevalence of unilateral hearing loss was more prevalent in the 50–59 years old group, affecting 1.9% (95% CI, 1.3–2.6%) of subjects, as compared with 0.4% (95% CI, 0.0–0.9%) of subjects in the over 80 years old group; the prevalence across all age groups ranged from 0.4% to 1.9%. Mild hearing loss was the most common type of hearing loss in the 20–79 years old group, and the prevalence was the highest in the 70–79 years old group 44.6% (95% CI, 41.8–47.4%). In the over 80 years old group, the prevalence of moderate hearing loss (37.2% (95% CI, 33.7–40.8%)) exceeded that of mild hearing loss (36.7% (95% CI, 33.2–40.3%)).

### Hearing thresholds of different frequencies in the otologically normal population

**Fig. 1:**
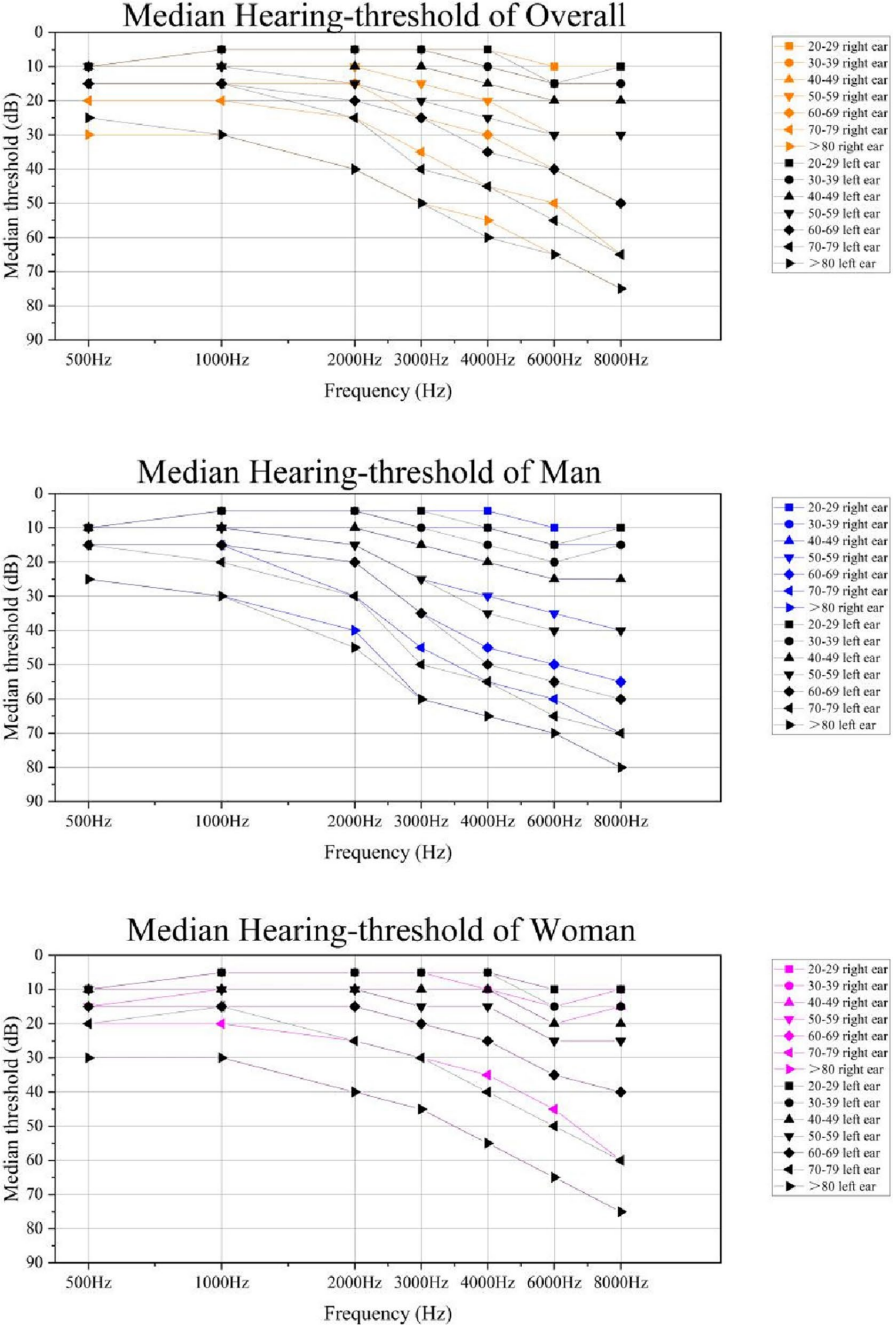
Median Hearing thresholds in dB as a function of frequencies at different ages Based on hearing threshold testing of National Health and Nutrition Examination Survey (NHANES) data from 1999–2018. Data represent estimated median of pure-tone thresholds assessed in the right and left ear among U.S. adults of overall, male and female, by seven frequencies (500, 1000, 2000, 3000, 4000, 6000, and 8000 Hz), NHANES1999-2018. Data were weighted to be nationally representative

Figure 1 shows the median of pure tone thresholds (right ear and left ear) presented by age.

Table 5 shows the pure tone thresholds (average of participant’s right ear and left ear) and the pure tone thresholds according to ISO 7029:2017 at the median, and the 10th, 25th, 75th, and 90th percentile values for men and women, according to age and frequency in the otologically normal population. Among the otologically normal population, hearing thresholds increased with age across the entire frequency range. Hearing thresholds for the otologically normal population tend to exceed the ISO age- and sex-adjusted thresholds. However, the hearing thresholds for the over 80 years old group were better than the theoretical ISO norm thresholds for 1000, 2000, 6000, and 8000 Hz.

**Table 5:** Hearing thresholds (in decibels): comparison of the otologically normal population stratified by age and sex with standard values given in ISO 7029:2017 (Acoustics – Statistical distribution of hearing thresholds related to age and gender)

Frequency (Hz)	Age (years)	Hearing threshold (dB)									
		Men					Women				
		*P*_10_	*P*_25_	*P*_50_	*P*_75_	*P*_90_	*P*_10_	*P*_25_	*P*_50_	*P*_75_	*P*_90_
500	20–29	0(-5)	2.5(-3)	5.0(0)	10.0(4)	15.0(8)	0(-5)	2.5(-2)	7.5(0)	10.0(4)	15.0(7)
	30–39	0(-5)	2.5(-3)	7.5(0)	12.5(4)	17.5(8)	0(-6)	2.5(-3)	7.5(0)	10.0(4)	15.0(8)
	40–49	0(-5)	2.5(-2)	7.5(1)	10.0(4)	15.0(6)	0(-5)	2.5(-2)	7.5(1)	12.5(5)	17.5(8)
	50–59	0(-4)	5.0(-1)	7.5(3)	12.5(6)	20.0(10)	2.5(-3)	7.5(0)	10.0(3)	15.0(7)	22.5(11)
	60–69	2.5(-2)	5.0(2)	10.0(6)	17.5(11)	25.0(16)	2.5(0)	7.5(3)	12.5(6)	17.5(12)	27.5(16)
	70–79	5.0(3)	7.5(6)	15.0(10)	22.5(17)	22.5(23)	5.0(3)	10.0(7)	12.5(12)	25.0(18)	32.5(24)
	> 80	15.0(8)	15.0(12)	15.0(16)	27.5(24)	35.0(32)	12.5(6)	22.5(12)	22.5(19)	30.0(27)	35.0(35)
1000	20–29	0(-6)	2.5(-3)	5.0(0)	7.5(4)	12.5(7)	0(-5)	0(-3)	2.5(0)	5.0(3)	10.0(7)
	30–39	0(-6)	2.5(-3)	7.5(0)	10.0(4)	15.0(8)	0(-6)	2.5(-3)	5.0(0)	10.0(4)	15.0(8)
	40–49	0(-4)	5.0(-1)	7.5(2)	12.5(5)	20.0(8)	2.5(-5)	5.0(-2)	7.5(1)	12.5(5)	17.5(8)
	50–59	2.5(-3)	7.5(0)	10.0(4)	15.0(9)	22.5(13)	2.5(-3)	7.5(0)	10.0(4)	12.5(8)	20.0(12)
	60–69	5.0(-1)	7.5(3)	12.5(8)	20.0(15)	25.0(21)	5.0(1)	7.5(4)	12.5(8)	17.5(13)	30.0(19)
	70–79	5.0(4)	15.0(8)	17.5(13)	22.5(22)	30.0(29)	7.5(5)	12.5(9)	15.0(14)	25.0(22)	37.5(28)
	> 80	20.0(11)	20.0(15)	20.0(21)	22.5(29)	37.5(37)	15.0(11)	15.0(17)	20.0(24)	35.0(32)	37.5(40)
2000	20–29	-2.5(-6)	0(-3)	2.5(0)	7.5(3)	12.5(7)	-2.5(-5)	0(-3)	2.5(0)	7.5(3)	12.5(6)
	30–39	-2.5(-6)	0(-3)	5.0(1)	10.0(4)	17.5(8)	0(-6)	2.5(-3)	5.0(0)	10.0(4)	15.0(7)
	40–49	0.0(-3)	2.5(0)	7.5(3)	12.5(7)	20.0(11)	0(-5)	2.5(-2)	7.5(2)	12.5(5)	17.5(9)
	50–59	2.5(-1)	5.0(2)	12.5(6)	17.5(13)	27.5(19)	2.5(-2)	5.0(1)	10.0(5)	15.0(10)	20.0(14)
	60–69	2.5(2)	7.5(7)	15.0(12)	27.5(22)	35.0(30)	5.0(2)	7.5(6)	12.5(10)	22.5(17)	30.0(24)
	70–79	12.5(8)	17.5(14)	25.0(21)	60.0(31)	60.0(39)	5.0(7)	12.5(13)	20.0(19)	35.0(28)	40.0(35)
	> 80	32.5(18)	32.5(24)	40.0(32)	40.0(40)	40.0(47)	12.5(17)	22.5(24)	27.5(32)	40.0(41)	52.5(49)
3000	20–29	0(-6)	2.5(-3)	5.0(0)	10.0(3)	15.0(7)	-2.5(-6)	0(-3)	2.5(0)	7.5(3)	10.0(7)
	30–39	0(-6)	2.5(-3)	7.5(1)	12.5(5)	20.0(8)	-2.5(-7)	0(-3)	5.0(0)	10.0(4)	15.0(8)
	40–49	2.5(-3)	7.5(0)	12.5(4)	20.0(9)	32.5(13)	0(-4)	5.0(-1)	7.5(2)	12.5(6)	20.0(10)
	50–59	7.5(0)	12.5(4)	17.5(9)	27.5(17)	42.5(24)	2.5(-1)	7.5(2)	12.5(6)	17.5(12)	25.0(17)
	60–69	10.0(5)	20.0(10)	27.5(17)	40.0(27)	55.0(37)	5.0(3)	10.0(8)	17.5(13)	25.0(21)	37.5(28)
	70–79	15.0(12)	22.5(19)	27.5(27)	62.5(38)	62.5(48)	12.5(10)	17.5(16)	27.5(23)	42.5(33)	47.5(41)
	> 80	37.5(25)	37.5(33)	52.5(41)	52.5(47)	55.0(53)	17.5(22)	27.5(30)	40.0(38)	45.0(48)	57.5(57)
4000	20–29	-2.5(-6)	2.5(-3)	5.0(0)	10.0(4)	15.0(7)	-5(-6)	0(-3)	5.0(0)	7.5(4)	12.5(7)
	30–39	0(-6)	5.0(-2)	10.0(1)	17.5(5)	25.0(9)	-2.5(-7)	2.5(-3)	5.0(1)	12.5(5)	15.0(8)
	40–49	7.5(-2)	10.0(1)	15.0(4)	25.0(10)	37.5(15)	2.5(-4)	5.0(-1)	10.0(3)	15.0(7)	22.5(11)
	50–59	7.5(2)	15.0(6)	22.5(11)	35.0(20)	50.0(28)	5.0(-1)	10.0(3)	12.5(7)	20.0(14)	27.5(19)
	60–69	12.5(7)	25.0(13)	35.0(20)	50.0(32)	67.5(43)	7.5(4)	12.5(9)	20.0(15)	30.0(24)	45.0(32)
	70–79	22.5(16)	32.5(24)	40.0(33)	65.0(45)	65.0(55)	17.5(12)	25.0(19)	47.5(27)	52.5(37)	62.5(46)
	> 80	50.0(32)	50.0(40)	55.0(50)	55.0(55)	60.0(59)	25.0(27)	35.0(35)	40.0(43)	52.5(53)	67.5(62)
6000	20–29	2.5(-6)	7.5(-3)	12.5(0)	17.5(4)	22.5(8)	2.5(-6)	7.5(-3)	10.0(0)	17.5(4)	22.5(8)
	30–39	5(-6)	7.5(-2)	15.0(1)	20.0(6)	30.0(11)	5.0(-7)	10.0(-3)	12.5(1)	17.5(6)	25.0(10)
	40–49	10(-2)	15.0(2)	22.5(6)	27.5(13)	37.5(19)	7.5(-4)	12.5(-1)	17.5(4)	25.0(9)	32.5(14)
	50–59	15(3)	20.0(8)	25.0(14)	42.5(25)	57.5(35)	12.5(-1)	15.0(4)	22.5(10)	30.0(17)	40.0(24)
	60–69	17.5(10)	32.5(18)	45.0(26)	60.0(40)	72.5(53)	15.0(5)	20.0(12)	27.5(19)	42.5(30)	57.5(39)
	70–79	35(22)	35.0(32)	55.0(43)	62.5(55)	70.0(66)	17.5(15)	25.0(24)	45.0(33)	52.5(45)	65.0(56)
	> 80	57.5(43)	57.5(53)	60.0(64)	60.0(66)	87.5(69)	27.5(35)	42.5(43)	47.5(52)	70.0(62)	72.5(72)
8000	20–29	2.5(-6)	5.0(-3)	10.0(0)	15.0(5)	22.5(9)	2.5(-6)	7.5(-3)	10.0(0)	17.5(5)	20.0(9)
	30–39	5(-6)	7.5(-2)	12.5(2)	20.0(7)	27.5(12)	5.0(-7)	10.0(-3)	12.5(1)	20.0(7)	25.0(11)
	40–49	10(-2)	15.0(2)	22.5(7)	32.5(15)	40.0(22)	7.5(-4)	12.5(0)	17.5(5)	25.0(11)	35.0(17)
	50–59	17.5(4)	25.0(10)	32.5(16)	45.0(29)	65.0(40)	12.5(0)	17.5(6)	25.0(12)	35.0(21)	45.0(29)
	60–69	20(13)	37.5(21)	57.5(30)	72.5(46)	85.0(60)	20.0(6)	25.0(14)	37.5(23)	52.5(35)	70.0(45)
	70–79	42.5(26)	57.5(37)	57.5(50)	65.0(63)	75.0(74)	22.5(18)	27.5(28)	52.5(39)	60.0(52)	70.0(63)
	> 80	62.5(44)	62.5(59)	65.0(75)	65.0(76)	77.5(77)	32.5(40)	37.5(49)	57.5(60)	67.5(70)	77.5(80)

() = ISO 7029:2017 (Acoustics – Statistical distribution of hearing thresholds related to age and gender)

The median values of hearing thresholds in men in over 80 years old group tended to be better than the ISO 7029:2017 values for 500, 1000, 6000, and 8000 Hz, whereas they were worse for very high frequencies.

Our results show lower median values across the entire frequency range (except for 500 and 3,000 Hz) for women in over 80 years old group, compared with those given in ISO 7029:2017, Compared with the ISO 7029:2017 norms for women over 80 years old, the thresholds obtained in this study were better for 1000, 2000, 3000, 4000, 6000, and 8000 Hz but worse for 500 Hz.

## Discussion

In this study we used the temporal cross-sectional data from NHANES (1999 to 2018) to explore temporal trends in the prevalence of hearing loss among adults in the USA. It was found that, overall, there has been a statistically significant decrease in the prevalence of hearing loss among US adults over the years studied. However, the severity of hearing loss statistically significantly increased by cycle. Among US adults, mild hearing loss was more prevalent in those aged 20–79 years, while in those aged over 80 years the prevalence of moderate hearing loss exceeded that of mild loss. The prevalence of hearing loss also varied significantly by age group, sex, race, educational level, PIR, and noise exposure. Over the study period, the prevalence of hearing loss increased significantly among women, non-Hispanic whites, those with high school and college or higher educational levels, those with a middle or high PIR, and those experiencing non-occupational noise exposure.

While the prevalence of hearing loss in US adults showed a decreasing trend, the severity of hearing loss increased by cycles. A number of factors could account for the decrease in the prevalence of hearing loss, including demographic shifts, lifestyle changes, increased awareness of using hearing protection, increased noise control measures, and improvements in the accessibility and affordability of hearing care [7, 9, 12]. Hearing loss is a progressive process that, without appropriate intervention, gradually worsens over time. The lack of adequate coverage and the low uptake of hearing healthcare have contributed to the annual increase in the severity of hearing loss [15, 16].

We observed that age was significantly associated with the prevalence of hearing loss. In some age subgroups (30–39, 60–69, 70–79, and > 80 years), the prevalence of hearing loss decreased by cycle. In the present study, the prevalence of unilateral hearing loss did not simply increase with age, but increased up to a certain age group and then decreased. The prevalence of unilateral hearing loss increased from 20 to 59 years old, and decreased after age 60 years. This finding is consistent with reports in the current literature [17, 18]. The prevalence of age-related hearing loss (ARHL) would be expected to increase as the number of older Americans increases due to aging of the population. ARHL is not a single event but the result of multiple factors (such as genetic, biological, social, psychological, and environmental factors) experienced from prenatal to childhood and adulthood to old age. The opposite result observed in our study may be related to the delayed onset of ARHL due to the delay in the onset of chronic disease due to following a healthy lifestyle, which effectively reduces the burden of age-related disease [15]. In recent years, the risk of ARHL may have been greatly reduced by people making healthy lifestyle choices, such as following a good diet, engaging in physical activity, and avoiding smoking, in addition to engaging in preventive behaviors such as ensuring protection from loud noise [19].

In our study, the prevalence of hearing loss in men was higher than that in women, but it was increasing by cycle in women. Hearing loss in women may be related to decreased estrogen. Numerous studies have demonstrated that estrogen has a positive effect on hearing function [20], and the hearing threshold declines in post-menopausal women due to the decline in estrogen levels. Therefore, as the population ages, the gender difference between the prevalence of hearing loss will reduce, as the prevalence of hearing loss in women increases by cycle. Even though the prevalence of hearing loss in men shows no significant overall temporal trend, it is still a significant concern because the prevalence in men is higher than in women. Studies have shown that men are more likely to suffer from noise-induced hearing loss (NIHL) [21].

We also found in this study that non-Hispanic blacks have a lower prevalence of hearing loss than other racial groups, while non-Hispanic whites are more likely to have hearing loss. This finding is similar to the findings of some previous studies [11, 12, 22]. However, evidence from previous studies suggests that hearing loss is very common among black and Hispanic subgroups, including Mexicans and Cubans [23]. Their culture, perceptions concerning hearing protection, and factors associated with changing one’s behavior may contribute to differences in hearing loss between ethnicities.

The findings from our study suggested that the higher the level of education [10] the lower the likelihood that a person will experience hearing loss. This may be related to the fact that people with a higher educational level are more likely to have access to an environment with less noise exposure and more resources for hearing protection [24].

This study showed an increased prevalence of hearing loss in the group who experienced noise exposure. Noise exposure is an important factor in hearing loss, and is strongly related to sex and occupation [25], which may have contributed to the higher prevalence of hearing loss in men and those who experience occupational noise exposure. Although the preventive strategy and measurements made to prevent hearing loss due to exposure to noise have gradually improved in the USA, there is room for further improvement [26]. The increase in hearing loss due to occupational noise exposure may be related to existing regulations for noise in the workplace, which strike a balance between hearing protection and economic development, underestimate the adverse health and social costs of noise-induced hearing loss, and do not fully protect workers from hearing injury [27]. On the other hand, as an economy develops, people have more opportunities and time to be exposed to recreational noise. Although noise control policies and regulations in entertainment venues have been developed to help people avoid exposure to loud noise [28, 29], the increasing prevalence of hearing loss associated with non-occupational noise exposure indicates that more effective measures regarding noise control and hearing protection are needed.

Hearing thresholds for otologically normal population tend to exceed the ISO 7029:2017 age- and sex-adjusted hearing thresholds. However, with respect to the percentile values of hearing threshold distributions observed in the otologically normal population, hearing sensitivity was found to be less pronounced in the over 80 years old group at all frequencies, except for 500 kHz, than as described in ISO 7029:2017. Similar findings have been reported in some other studies [30, 31]. This finding may be related to genetic disposition and the change of lifestyle.

The findings of our study have important public health implications. To reduce the growing burden of hearing loss in the USA, better integrated interventions for hearing healthcare need to be developed through periodic evaluation of the trend in hearing loss and a better understanding of the risk factors for hearing loss. The epidemiological information provided by this study comprehensively illustrate the change in the prevalence of hearing loss, and provide an understanding of the developing trend of hearing loss. Our study may provide valuable insights for those who need to understand the hearing needs of a population, make public health plans related to hearing, and make policy decisions and allocate resources for hearing care. The results of this study show that the hearing threshold levels for each frequency have changed. Our data can provide a reference for updating the ISO 7029:2017 age- and sex-adjusted hearing thresholds, in order to bring the standard more in line with the real-world situation in the population.

In this study we analyzed the nationally representative auditory data for 1999–2018 from NHANES to calculate the prevalence of and estimate the trends in hearing loss among adults. Few prior studies have evaluated hearing-loss trends over such an extended period. Furthermore, in NHANES, hearing loss was assessed using the gold standard objective audiometric measurement, which has been proven to be reliable in many studies. However, the limitations of this study should be considered. First, this study was cross-sectional and therefore could not examine the causality of the association of potential risk factors with hearing loss. Moreover, because of the NHANES study design, the age of the study participants was not continuous in the cycle, which was not conducive to extrapolation, and therefore more studies are needed that involve carrying out this hearing test on the same age group. Finally, Selective bias has been addressed as far as possible by adjusting the weights to make the population representative. Measurement error caused by the change of the measurement instrument in audiometric methods is likely to exist.

## Conclusions

In this survey study, the estimated prevalence of hearing loss decreased from 1999 to 2018; the prevalence among those aged 60 years or older remained high but decreased. Mild hearing loss was more prevalent among US adults aged 20–79 years, while in those aged over 80 years the prevalence of moderate hearing loss exceeded that of mild loss. We observed that hearing thresholds tend to exceed the ISO 7029:2017 age- and sex-adjusted hearing thresholds for the otologically normal population, while the percentile of the hearing threshold distribution for people over 80 years old is lower than given in ISO 7029:2017. It is of great importance to continue to monitor hearing threshold distributions in the elderly, the trends in hearing loss, and the factors that influence hearing loss in the same age groups looked at in this study.

## Data Availability

The datasets analyzed during the current study are available in the NHANES repository, https://wwwn.cdc.gov/nchs/nhanes/Default.aspx.

## Abbreviations

U.S.: United States
GBD: Global Burden of Disease Study
YLDs: Years lived with disability
WHO: World Health Organization
NHANES: National Health and Nutrition Examination Survey
CDC: Center for Health Statistics ant the Centers for Disease Control and Prevention
NIOSH: National Institute for Occupational Safety and Health
MEC: Mobile Examination Center
PTA: Pure-tone average
PIR: Poverty/income ratio
ARHL: Age-related hearing loss
NIHL: Noise-induced hearing loss
P10: 10Th percentile
P25: 25Th percentile
P50: 50Th percentile
P75: 75Th percentile
P90: 90Th percentile
